# Bilateral Infectious Extensor Tenosynovitis: A Case Report

**DOI:** 10.5811/cpcem.1317

**Published:** 2023-04-18

**Authors:** Daria Osipchuk, Jeffrey Riddell

**Affiliations:** Keck School of Medicine of University of Southern California, Department of Emergency Medicine, Los Angeles, California

**Keywords:** case report, extensor tenosynovitis

## Abstract

**Introduction:**

Infectious extensor tenosynovitis is a rare infection spreading along the extensor tendons of the extremities. It presents a diagnostic challenge in the emergency department (ED) given the nonspecific signs and symptoms, as opposed to the more common flexor tenosynovitis that is diagnosed by the classic Kanavel signs on physical exam.

**Case Report:**

Here we present a case of bilateral extensor tenosynovitis in a 52-year-old female denying past medical history who presented to the ED with two days of bilateral dorsal hand swelling and pain. She denied any risk factors such as direct trauma to the hands or intravenous drug use. The rare diagnosis was suspected in the ED due to a very high complement reactive protein level and a concerning point-of-care ultrasound. Extensor tenosynovitis was ultimately confirmed on computed tomography and by operative irrigation and drainage of the tendon sheaths.

**Conclusion:**

This case demonstrates the importance of keeping extensor tenosynovitis on the differential when seeing a patient with dorsal extremity edema and pain, even if the findings occur bilaterally.

## INTRODUCTION

Purulent tenosynovitis is an infection that tracks along tendons. Most commonly it is seen as a flexor tenosynovitis of the hand and wrist, identified by the Kanavel signs, which include pain with palpation along the flexor tendon, the digit held in flexion, pain with passive extension of the digit, and fusiform swelling of the digit.^1^ It is much rarer to find in extensor tendons, with only a few case reports and case series documenting infectious extensor tenosynovitis in the literature. The findings in extensor tenosynovitis lack the classic characteristic Kanavel signs of flexor tenosynovitis and, thus, there are frequent delays in diagnosis. As with most hand infections, patients usually report trauma or preceding intravenous (IV) drug use, and the classic dogma is that infections occur unilaterally.^2^,^3^ Here we report a case of bilateral extensor tenosynovitis in a patient denying direct trauma or IV drug use.

## CASE REPORT

A 52-year-old female with no past medical history presented to the ED with bilateral hand swelling and pain for two days. The patient reported that a few days prior she had strained her left shoulder while holding a rope to help somebody cut down a tree. She presented to an outside hospital, was diagnosed with a rotator cuff tear, and was given IV morphine in the left upper extremity. Subsequently, she developed left hand swelling. She went back to the outside hospital, received IV morphine in the right upper extremity, and developed right hand swelling. The patient denied any direct trauma to her hands. The pain was only alleviated by placing her hands in cold water. In addition, she endorsed atraumatic left knee swelling. The patient endorsed smoking marijuana and a remote history of methamphetamine use but denied any current tobacco, alcohol, or IV drug use.

On arrival, the patient was afebrile with an oral temperature of 36.6° Celsius, tachycardic to 110 beats per minute, and had a blood pressure of 142/106 millimeters of mercury. Exam revealed puffy edema on the dorsal side of the bilateral hands extending to the wrists with mild erythema bilaterally over the dorsum without overlying induration or fluctuance. Range of motion of fingers and wrists was intact bilaterally but somewhat limited by swelling. There was no pain with micromotion of the wrists. Sensation was intact with strong bilateral radial pulses, brisk capillary refill, and soft compartments. Exam of the left knee revealed mild erythema and edema over the distal thigh with warmth over the knee. There was full range of motion of the knee but pain at extremes of motion. The dorsalis pedis pulse was strong, and the compartments were soft.

Initial differential diagnosis included rheumatologic conditions such as rheumatoid arthritis and systemic lupus erythematosus, infectious etiologies, and allergic reactions to receiving IV morphine. Given the multiple sites of arthralgias without any direct trauma, and minimal erythema with intact range of motion, rheumatologic conditions were at the top of the differential. The appearance of the edema without significant erythema or induration was less consistent with cellulitis. Intact range of motion with arthralgias in multiple locations pointed away from septic arthritis in a patient without a history of IV drug use. Necrotizing soft tissue infection was also unlikely given absence of fever, pain out of proportion, crepitus, or bullae.

Laboratory studies revealed a white blood cell count of 20,000 per millimeter^3^ (K/mm^3^) (reference range: 4.5–10 K/mm^3^), 22% bands (0–9%), complement reactive protein (CRP) 631.2 milligrams/liter (mg/L) (0–4.9 mg/L), and rheumatoid factor 45 international units/milliliter (IU/mL) (0–13 IU/mL). Radiographs of the bilateral hands and wrists revealed no acute fracture or dislocation but showed diffuse soft tissue swelling.

Given the laboratory studies of leukocytosis, bandemia, and a very elevated CRP, IV ceftriaxone and vancomycin were started out of concern for bacterial infection. Point-of-care ultrasound was performed, which demonstrated bilateral wrist effusions and fluid surrounding the extensor tendon sheaths.

Orthopedic surgery was consulted and performed bilateral wrist arthrocentesis with aspiration of purulent fluid. Synovial fluid analysis for the left and right wrist demonstrated nucleated cell counts of 37,200 cells/microliter (cells/μL) (reference range: 13–180 cells/μL) and 36,850 cells/μL, and percentage segmented neutrophils of 92% (reference 0–25%) and 98%, respectively. Arthrocentesis of the left knee was also performed, and synovial fluid analysis revealed a nucleated cell count of 24,000 cells/μL and percentage segmented neutrophils of 90%. Computed tomography (CT) of the bilateral upper extremities showed bilateral extensor tenosynovitis and multiple abscesses on the dorsal hands.

The patient was admitted to the inpatient service for extensor tenosynovitis and septic arthritis and ultimately was taken to the operating room by orthopedic surgery. Operative findings showed left extensor tenosynovitis in hand compartments three and four and right extensor tenosynovitis in compartments two, three, and four with tenosynovectomies performed. The bilateral septic wrist joints and left septic knee joint were irrigated and debrided. Wound cultures and blood cultures showed no growth of either bacteria or fungus. The *Neisseria gonorrhea* test came back negative. Inpatient broad-spectrum antibiotics were continued, and she was discharged on hospital day 10 with a three-week course of trimethoprim/sulfamethoxazole.

*CPC-EM Capsule*

What do we already know about this clinical entity?

*Infectious extensor tenosynovitis is usually a unilateral infection that can be identified by pain, edema, and erythema on the dorsum of the extremity*.

What makes this presentation of disease reportable?

*This rare case of extensor tenosynovitis occurred in the bilateral hands without inciting trauma*.

What is the major learning point?

*Point-of-care ultrasound can aid in the diagnosis while awaiting computed tomography, magnetic resonance imaging, or the gold standard of surgical debridement*.

How might this improve emergency medicine practice?

*Extensor tenosynovitis should be on the differential for anyone presenting with soft tissue edema of the dorsum of the hand, even if findings are bilateral*.

## DISCUSSION

This is a rare case of extensor tenosynovitis, even more unusual given it occurred bilaterally without inciting trauma. Tenosynovitis occurs when an infectious or inflammatory fluid fills a potential space in the tendon sheath between the visceral and parietal layers.^4^ Infection is usually introduced by direct inoculation by penetrating trauma or IV drug use and can spread through the parietal tendon sheaths to surrounding structures.^3^–^5^ The extensor tendons are less isolated than flexor tendons; so infection is less likely to be localized exclusively to the extensor tendons.^2^ Findings of extensor tenosynovitis can be nonspecific but include pain, edema, and erythema on the dorsum of the extremity.^2^,^5^

Point-of-care ultrasound (POCUS) may be used to aid in the diagnosis of extensor tenosynovitis by extrapolating from studies on flexor tenosynovitis. Ultrasound has high sensitivity for the diagnosis of flexor tenosynovitis when compared to the gold standard of intraoperative findings during surgical debridement.^6^ Findings indicating tenosynovitis include hypoechoic or anechoic effusions surrounding the tendons without Doppler flow and thickening of the synovial sheath.^7^ When performing ultrasound, it can be useful to compare to an unaffected digit for a healthy tissue comparison.^6^

While bilateral infections are uncommon, polyarthralgia secondary to disseminated bacteremia in conditions such as endocarditis or disseminated gonorrhea does occur.^8^,^9^ This patient, however, tested negative for *N. gonorrhea*. Unfortunately, she did not have an echocardiogram done prior to discharge, but without a history of IV drug use and with negative blood cultures, this would also be unlikely. She did receive IV morphine in her bilateral arms shortly prior to developing hand edema, but it would be unusual for an IV line placed in a hospital setting and removed shortly thereafter to seed an infection significant enough to cause bilateral tenosynovitis and septic arthritis.

Given that this patient’s physical exam findings were nonspecific, the laboratory studies in this case, along with the POCUS, were key in making the diagnosis. Particularly, the very elevated CRP of 631.2 mg/L made it likely that this patient had a bacterial infection. In one study, rheumatologic conditions typically had a CRP level less than 250 mg/L, and 88.9% of cases of high CRP greater than 350mg/L were attributed to infection.^10^ In another study, 88% of patients with a CRP greater than 500 mg/L were found to have an infection, and their mortality rate was 36%.^11^

Even with a very high CRP, distinguishing inflammatory versus infectious tenosynovitis can be challenging. Obtaining a good history can be useful, with a history of trauma to the extremity suggesting an infectious etiology, whereas a history of autoimmune disease or prior episodes of joint or extremity swelling would suggest an inflammatory etiology. Inflammatory tenosynovitis may also occur from overuse syndromes, gout, or pseudogout.^12^–^14^ In addition to ultrasound, CT and magnetic resonance imaging (MRI) can demonstrate tenosynovitis and often demonstrate abscesses adjacent to the extensor tendons, which would be more suggestive of a septic etiology.^3^ Operative debridement is the gold standard. In this case, the intraoperative findings of purulent fluid in the synovial sheaths confirmed the diagnosis.

Limitations of this case report include that this patient was treated at an outside hospital prior to her arrival in our ED. She reported receiving IV morphine in her bilateral upper extremities at the outside hospital in the days prior to her bilateral hand swelling, but we cannot confirm where the IVs were placed in relation to where the infections occurred and whether this could have represented a form of direct trauma to inoculate the infection into the dorsum of her hands.

## CONCLUSION

Extensor tenosynovitis should be kept high on the differential for anyone presenting with soft tissue edema of the dorsum of the hand. While bilateral infections are rare, they do occur. Point-of-care ultrasound can help with making the diagnosis of extensor tenosynovitis while awaiting CT, MRI, or surgical consult. Any patient with an extremely high CRP greater than 350 mg/L should be presumed to have an infection until proven otherwise and started on antibiotics quickly, particularly as the mortality rate associated with these CRP levels is high. The gold standard for diagnosis and treatment of extensor tenosynovitis is surgical irrigation and debridement.
